# Screening eating disorders among laboratory workers

**DOI:** 10.1192/j.eurpsy.2026.11365

**Published:** 2026-07-31

**Authors:** I. Sellami, A. Abbes, A. Feki, D. Elleuch, M. L. Masmoudi, M. Hajjaji, K. Jmal Hammami

**Affiliations:** 1 occupational medicine; 2 Rheumatoloy, Hedi Chaker Hospital; 3 Family department, Medecine university, sfax, Tunisia

## Abstract

**Introduction:**

Eating disorders (EDs) are associated with substantial impairments in both physical and mental health. In the workplace, these conditions can adversely influence laboratory workers’ health and productivity.

**Objectives:**

This study aimed to screen EDs and mental health among laboratory workers.

**Methods:**

A descriptive, analytical, cross-sectional survey was carried out among laboratory workers. Data were gathered on sociodemographic and occupational characteristics. EDs were screened using the SCOFF questionnaire (Sick, Control, One stone, Fat, Food), while perceived stress was evaluated with the 4-item Perceived Stress Scale (PSS-4). Motivation for healthy eating was rated on a 0–10 scale, with 0 representing low and 10 representing high motivation.

**Results:**

The study population consisted of 91 laboratory workers, with a mean age of 44.2 ± 9.2 years. Among them, 83 (92.2%) were women, and 80% were married. The mean of tenure of job was 14.8 ± 9.5 years. The mean of the SCOFF score was 1.2 ± 1.3. We found that 37.8% of participants had possible EDs. The mean of the motivation for healthy eating was 7.1 ±2.3. The mean of perceived stress was 6.3 ± 2.6. The probability of EDs was higher in stressed laboratory workers (p =0.008).

**Conclusions:**

Eating disorders are common among laboratory workers. Our results also underscore the impact of stress on workers’ dietary habits. Raising awareness about the importance of a balanced and healthy diet is essential to support their mental well-being and, consequently, enhance workplace productivity.

**Disclosure of Interest:**

None Declared

